# A78 INTESTINAL AND PANCREATIC HNF4A ACTING TOGETHER TO MANAGE BLOOD GLUCOSE LEVELS

**DOI:** 10.1093/jcag/gwae059.078

**Published:** 2025-02-10

**Authors:** N Rodriguez Rodriguez, A de Castro, C Jones, D Pupo, F Boudreau

**Affiliations:** Universite de Sherbrooke Faculte de Medecine et des Sciences de la Sante, Sherbrooke, QC, Canada; Universite de Sherbrooke Faculte de Medecine et des Sciences de la Sante, Sherbrooke, QC, Canada; Universite de Sherbrooke Faculte de Medecine et des Sciences de la Sante, Sherbrooke, QC, Canada; Universite de Sherbrooke Faculte de Medecine et des Sciences de la Sante, Sherbrooke, QC, Canada; Universite de Sherbrooke Faculte de Medecine et des Sciences de la Sante, Sherbrooke, QC, Canada

## Abstract

NOT PUBLISHED AT AUTHOR’S REQUEST

Please acknowledge all funding agencies listed below

**Funding Agencies:** Bourses d’excellence de l’Université de Sherbrooke

